# *VARS2*-linked mitochondrial encephalopathy: two case reports enlarging the clinical phenotype

**DOI:** 10.1186/s12881-019-0798-7

**Published:** 2019-05-07

**Authors:** Chiara Begliuomini, Giorgio Magli, Maja Di Rocco, Filippo M. Santorelli, Denise Cassandrini, Claudia Nesti, Federica Deodato, Daria Diodato, Susanna Casellato, Delia M. Simula, Veronica Dessì, Anna Eusebi, Alessandra Carta, Stefano Sotgiu

**Affiliations:** 1 Unit of Child Neuropsychiatry Residency Program, University Hospital of Sassari, Viale San Pietro 43/B, I-07100 Sassari, Italy; 2 Department of Pediatrics, Unit of Rare Diseases, Giannina Gaslini Institute, Via Gerolamo Gaslini, 5, 16147 Genoa, Italy; 3 Molecular Medicine for Neurodegenerative and Neuromuscular Diseases Unit, IRCCS Fondazione Stella Maris, Viale del Tirreno, 331 56018 Calambrone, Pisa, Italy; 4 Metabolic Division, ‘Bambino Gesu’ Children’s Research Hospital, Piazza di Sant’Onofrio4, 00165 Rome, Italy; 5 Unit of Neuromuscular and Neurodegenerative Disorders, Laboratory of Molecular Medicine, ‘Bambino Gesu’ Children’s Research Hospital, Piazza di Sant’Onofrio, 4, 00165 Rome, Italy; 6 Child Psychiatry Unit, Department of Neuroscience, ‘Bambino Gesù’ Children’s Research Hospital, Piazza di Sant’Onofrio, 4, 00165 Rome, Italy

**Keywords:** Mitochondrial disorder, Epileptic encephalopathy, Developmental delay, VARS2

## Abstract

**Background:**

Mitochondrial respiratory chain consists of five complexes encoded by nuclear and mitochondrial genomes. Mitochondrial aminoacyl-tRNA synthetases are key enzymes in the synthesis of such complexes. Bi-allelic variants of *VARS2*, a nuclear gene encoding for valyl-tRNA (Val-tRNA) synthetase, are associated to several forms of mitochondrial encephalopathies or cardiomyoencephalopathies. Among these, the rare homozygous c.1100C > T (p.Thr367Ile) mutation variably presents with progressive developmental delay, axial hypotonia, limbs spasticity, drug-resistant epilepsy leading, in some cases, to premature death. Yet only six cases, of which three are siblings, harbouring this homozygous mutation have been described worldwide.

**Case presentation:**

Hereby, we report two additional cases of two non-related young girls from Sardinia, born from non-consanguineous and healthy parents, carrying the aforesaid homozygous *VARS2* variant. At onset both the patients presented with worsening psychomotor delay, muscle hypotonia and brisk tendon reflexes. Standard genetic tests were normal, as well as metabolic investigations. Brain MRI showed unspecific progressive abnormalities, such as corpus callosum hypoplasia (patient A) and cerebellar atrophy (patient A and B). Diagnosis was reached by adopting massive parallel next generation sequencing.

Notably clinical phenotype of the first patient appears to be milder compared to previous known cases. The second patient eventually developed refractory epilepsy and currently presents with severe global impairment. Because no specific treatment is available as yet, both patients are treated with supporting antioxidant compounds along with symptomatic therapies.

**Conclusions:**

Given the paucity of clinical data about this very rare mitochondrial encephalopathy, our report might contribute to broaden the phenotypic spectrum of the disorder. Moreover, noteworthy, three out of five pedigrees so far described belong to the Northern Sardinia ethnicity.

## Background

Mitochondrial protein synthesis involves an intricate interplay between mtDNA-encoded RNAs and nuclear DNA-encoded proteins, such as elongation factors, ribosomal proteins and aminoacyl-tRNA synthases. Among the 17 mitochondria-specific aminoacyl-tRNA synthases, *VARS2* encodes the mitochondrial valyl-tRNA synthase (mtValRS), a class I enzyme catalyzing the attachment of valine to its cognate tRNA molecule in a highly specific reaction [9].

Rare bi-allelic variants in *VARS2* have been associated with mitochondrial encephalopathies or cardiomyoencephalopathies in 13 families with 17 affected individuals worldwide [2, 6–8, 13]. To date the p.Thr367Ile variant is the most common. The homozygous c.1100C > T (p.Thr367Ile) mutation has been described in six patients presenting with encephalopathy [3, 6, 8] and the correlation between genotype and phenotype appears loose.

We present two further apparently unrelated children harbouring the homozygous c.1100C > T (p.Thr367Ile) mutation in *VARS2*, comparing their clinical features with previously reported patients and discussing on the unusual recurrence of this specific variant in northern Sardinia.

## Case report A

This is a 6-year old girl, second daughter from non-consanguineous and healthy parents. She was born by programmed caesarean delivery at 38 weeks of gestation, after an uneventful pregnancy. Birth parameters were normal: weight 97th, length 54th, head circumference 80th percentile. Postnatal adaptation was normal; APGAR scores were 9 and 10. Congenital hip dysplasia was treated with a harness. She was first referred to medical attention at 11 months for a progressive delay of psychomotor milestones. Neurological examination confirmed motor and language delays with only head control achieved, impaired social interaction, muscle hypotonia and brisk tendon reflexes. Routine blood test including thyroid hormones, serum creatine kinase, standard karyotype, cardiological and abdominal ultrasound evaluation were all normal. Routine EEG revealed increased background theta-delta activity with posterior spike-like elements, particularly in the right occipital area.

At 16 months, after global psychomotor training, social skills and language had improved, though she was not able to sit unsupported. Mild facial dysmorphic features were also noticed (hypertelorism, epicanthal folds, depressed nasal bridge, puffy hands and feet). Neurophysiological examination and laboratory investigations, including organic aciduria, serum aminoacid and lactate levels, were unremarkable.

At 32 months, developmental delay, axial hypotonia with limb hypertonia, brisk tendon reflexes and ankle hypomobility were prominent features with evidence of further regression. Brain MRI showed corpus callosum hypoplasia and cerebellar cortex atrophy (not shown). Array CGH and serial metabolic screening resulted normal.

**Fig. 1:**
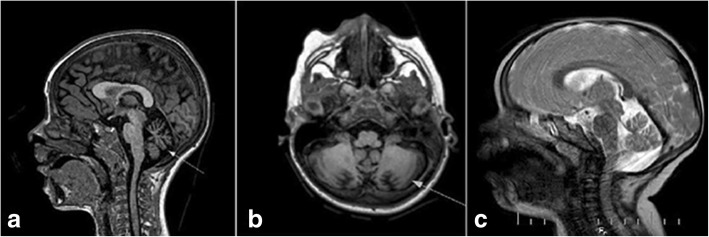
Patient A. Follow-up MRI at 47 months: **a**) Sagittal T1-weighted image showing cerebellar atrophy (arrow); **b**) Axial FLAIR image showing hyperintensity of cerebellar white matter and dentate nuclei (arrow). Patient B. MRI at 24 months: **c**) Sagittal T2-weighted image showing megacisterna magna and atrophy of vermis

Follow-up MRI at 47 months, revealed atrophic progression of the cerebellum with T2-FLAIR hyperintensities of cerebellar white matter and dentate nuclei (Fig. 1.a-b). MR spectroscopy of corresponding cerebellar white matter alterations showed increased lactate and decreased N-acetilaspartate peaks. Head circumference decreased up to the 10th percentile. Despite no seizures had ever been reported by parents or observed during hospitalizations, standard and sleep-deprived EEGs were significant for spike-waves involving temporal and occipital areas.

At about 5 years, neurological condition worsened. The patient could barely speak in sentences with simple words, had a limited broad-based gait with bilateral support; bilateral spasticity in upper and lower limbs was present. A skeletal muscle biopsy revealed partial reduction of respiratory chain enzyme complexes I + III (0.13 mmol/min/gr of tissue, normal range 0.65–1.50). Blood DNA from the proposita was analysed using a customized targeted resequencing panel (MitoChip) able to investigate the coding regions of 1172 nuclear genes encoding the “MitoExome” [5]. Using this strategy we identified the homozygous c.1100C > T (p.Thr367Ile) in *VARS2* (NM_001167734). The mutation was confirmed by Sanger sequencing and segregated in heterozygosity in healthy parents.

At last follow up (age 6 years), the patient manifested sporadic and transient myoclonic movements of the right arm, proven to have a subcortical origin by EEG recording. She is currently able to walk with bimanual support and to formulate complex sentences; neither seizures nor dysphagia are reported. Myoclonic jerks are rare. She is attending school with good social skills. Moreover she is undergoing daily global psychomotor training. She is currently under Coenzyme Q10 (11 mg/kg/day) and Riboflavin (9 mg/kg/day) supportive antioxidant therapies.

## Case report B

This is a 5-year old girl, the only child from non-consanguineous and healthy parents. She was born after 41 weeks of uneventful gestation by natural delivery. Postnatal adaptation was normal. Due to congenital hip dysplasia, she was treated with a harness. At 12 months, the patient could not walk autonomously and was only able to sit unsupported. At 2 years, neurological examination revealed nystagmus with alternating strabismus, brisk tendon reflexes, global hypotonia and impaired coordination. Gait was possible only for a few steps with bimanual support; social skills and language appeared normal for age. Facial dysmorphisms were also recorded, and included microphthalmia, hypertelorism, strabismus, tilted ear axes and fleshy lips. Routine laboratory investigations in blood were uninformative, except for an increased serum lactate. Cardiological evaluation, abdominal ultrasound and genetic testing (standard karyotype, array CGH) were normal. Standard EEG showed modest non-epileptic abnormalities in both frontotemporal regions. Brain MRI at 2 years showed a mega-cisterna magna with signs of cerebellar atrophy (Fig. 1.c). At age 3, the patient underwent a global psychomotor training. After 9 months, she developed involuntary movements with recurrent paresis of the upper right limb. EEG showed sleep-driven spike-wave abnormalities in bilateral temporal regions. A successful treatment with Clobazam (10 mg/day) went on for 1 month.

Follow-up brain MRI performed at age 4, displayed cerebellar atrophy and vermis hypoplasia with normal spectroscopy (not shown). At 4.5 years, she complained of secondarily generalized tonic-clonic seizures with intensification of EEG epileptic abnormalities and was treated with Levetiracetam 300 mg/day (20 mg/kg). Severe psychomotor and social regression occurred. Exome sequencing using reported methodologies (Diodato D et al. 2014) revealed the homozygous c.1100C > T (p.Thr367Ile) mutation in VARS2. She is currently unable to sit and to speak. Due to swallowing difficulties she recently underwent percutaneous endoscopic gastrostomy. She is under antiepileptic polytherapy (Levetiracetam 20 mg/kg/day; Clonazepam 0.01 mg/kg/day) together with supportive antioxidant treatment (Q10 Coenzyme 11 mg/kg/day and Riboflavin 9 mg/kg/day).

## Discussion and conclusions

Owing to genetic and clinical heterogeneity, to nonspecific early signs and routine testing that are often uninformative, diagnosing mitochondrial diseases remains challenging [10]. We report two further children presenting *VARS2*-associated mitochondrial encephalopathy who had initially escaped correct diagnosis because of lack of specific metabolic indicators. When clinical and imaging findings were interpreted together with multigene sequencing, a formal diagnosis was finally formulated. Intriguingly, both patients harboured the same p.Thr367Ile mutation but formal relationship was denied.

As for clinical phenotype almost all patients with *VARS2* mutations present with severe early-onset encephalopathy with hypotonia. Albeit hypertrophic cardiomyopathy has never been observed in patients carrying the biallelic c.1100C > T (p.Thr367Ile) variant, this is a common feature of other known *VARS2* gene mutations, including those who are compound heterozygous for the p.Thr367Ile variant [3]. The early onset and the hypertrophic features suggest that cardiomyopathy could be related to a genetic etiology, rather than being a consequence of global hypotonia.

Furthermore, the biallelic c.1100C > T (p.Thr367Ile) variant seems to cover a heterogeneity spectrum, as follows:

Common features are very early microcephaly, hypotonia and global psychomotor delay, with the exception of our case A (normal head circumference at birth and whose microcephaly became evident only at the age 4; Table 1). Conversely, nystagmus (patients P3, P6 and case B), feeding difficulties (P3, P4, P6, case B) and limb spasticity (P6, P4, case A), are less common. Complications related to epilepsy, hypotonia and global debilitation are responsible for death, which occurred for P1, P3, P4 and P6 between 2 and 8 years. Seizures, ataxia and dystonic movements have their onset within the first years of life. Seizures almost invariably evolve to status epilepticus and refractory epilepsy from 2 to 4 years [3, 6, 8]. As it also occurred for our case B, seizures become the leading cause for clinical harmfulness. On the contrary, and despite the presence of epileptic EEG abnormalities, no seizure has been reported in our 6-year old girl (case A).

**Table 1:** Demographic and clinical review of the eight homozygous c.1100C > T, p. Thr367Ile VARS2 encephalopathy cases. Data on P1-P6 are obtained from literature (see references); cases A and B are described in present study

	Origin	Sex	First medical referral	Current age/death	Clinical and neurological signs	Lab tests	Brain MRI (spectroscopy)	OXPHOS study
P1^a1^	Italy (Sardinia)	M	First months	Death at 8 years	Developmental delay, microcephaly, seizures, nystagmus, facial dysmorphia	n.a.	T2 hyperintensity in insulae, right fronto-temporal cortex, periventricular WM (lactate in frontal WM)	Complex I deficiency (25% residual activity)
P2^2^	Poland	F	Birth	Alive at 5 years	Hypotonia, developmental delay, ataxia, seizures, pathological visual evoked potentials	n.a.	T2/FLAIR WM-hyperintensity, cerebral atrophy (lactate)	n.a.
P3^b2^	Afghanistan	F	6 months	Death at 7 years	Microcephaly, severe hypotonia, developmental delay, nystagmus, seizures	Serum lactate 2.3 mmol/l (normal 0.55–2.00)	Cerebellar atrophy, T2 hyperintensity of dentate nuclei & thalami; thin corpus callosum	n.a.
P4^b2^	Afghanistan	F	6 months	Death at 8 years	Progressive microcephaly, hypotonia, developmental delay, limb spasticity, seizures	Serum lactate 2.8 mmol/l (normal 0.55–2.00)	Cerebellar atrophy, T2 hyperintensity of dentate nuclei & thalami; thin corpus callosum	n.a.
P5^b2^	Afghanistan	M	Birth	Alive at 5 months	Hypotonia, progressive microcephaly	Serum lactate 4.4 mmol/l (normal 0.55–2.00) CSF lactate 3.08 mmol/l (normal 1.2–2.1)	Cerebellar hypoplasia	n.a.
P6^3^	Portugal	F	Birth	Death at 28 months	Developmental delay, nystagmus, severe hypotonia, spastic tetraparesis, microcephaly, seizures, feeding difficulties	Serum lactate 2.72 mmol/L (normal 0.55–2.00) CSF lactate 1.45 mmol/L (normal 1.2–2.1)	Global atrophy, diffuse T2 WM-hyperintensity	Complex II + III: 2.6 nmol/min/mg (normal 2.6–12)
A^4^	Italy (Sardinia)	F	11 months	6 years old	Motor and language delay, hypotonia, limb spasticity; dysmorphisms. No seizures	Normal serum lactate	T2 hyperintensity in deep WM and posterior internal capsule; corpus callosum hypoplasia and cortical cerebellar atrophy (increased lactate and decreased NAA in cerebellar WM lesions)	Complex I and III deficiency
B^4^	Italy (Sardinia)	F	2 years	5 years old	Developmental delay, nystagmus, hypotonia, limb spasticity; facial dysmorphisms; seizures	Slightly increased serum lactate	First MRI: mega-cisterna magna with slight cerebellar atrophy; cerebellar atrophy and vermis hypoplasia at follow-up MRI (normal spectroscopy)	n.a.

Legend: *M* male, *F* female, *WM* white matter, *NAA* N-acetyl-aspartate, *n.a*. not applicable

^a^ Clinical update on P1, subsequent to 2014 publication, was obtained from his medical records at the Unit of Child Neuropsychiatry, University Hospital of Sassari, where he has been treated

^b^Siblings

^1^ Diodato et al. [6]; ^2^Bruni et al. [3]; ^3^Pereira et al. [8]; ^4^Present study

Other shared clinical features relate to serial brain MRI showing increasing T2/FLAIR hyper-intensities in supra- and infratentorial regions and progressive cerebral volume loss. The occurrence of cerebellar atrophy does not come as a surprise in tRNA synthetase disorders but it appears relatively frequent in the cases harbouring the p.Thr367Ile mutation. When performed, MR-spectroscopy can either reveal lactate peaks (P1, P2 and case A) or appear uninformative even in severe cases (such as our case B).

Muscle OXPHOS activity is clinically less uniform and, when reported, shows either Complex I and combined Complex I and IV deficiencies (P1 and P6; Table 1) [3, 6] or combined Complex I and III activity as in our case A. OXPHOS was not investigated in case B.

Finally, definition of a molecular diagnosis in mitochondrial encephalopathies is not paralleled by more accurate therapies. Supportive antioxidant and bioenergetic compounds [4] combined with symptomatic therapies, remain the main option although apparently unable of arresting the clinical progression.

The p.Thr367Ile mutation appears to recur in *VARS2*-related encephalopathy in Sardinia. North Sardinians are an inbred and isolated population [1]. Selective centuries-old environmental pressures have also contributed to the high genetic peculiarity that renders Sardinians susceptible to several multifactorial conditions including multiple sclerosis and type 1 diabetes [11, 12]. We speculate that the high recurrence of *VARS2* related encephalopathy could be associated with a higher than expected rate of carriers, whose earlier identification would be fundamental to promote antenatal definition of genetic risk.

Three out of eight patients of literature originated from this area although the identical mutation occurs in other ethnicities, likely suggesting a mutational hot spot rather than a genetic drift. Despite the clinical phenotype of 2 out of 3 Sardinian cases do not substantially differ from that of other cases described, further investigations are recommended to define whether a shared haplotype underlies the three Sardinian cases.

## Abbreviations

Array CGH: Array - Comparative Genomic Hybridization
EEG: electroencephalogram
MR: magnetic resonance
MRI: magnetic resonance imaging
OXPHOS: OXydative PHOSphorilation
VARS: Valyl-tRNA synthetase
